# Mesenchymal Chondrosarcoma of Bone and Soft Tissue: A Systematic Review of 107 Patients in the Past 20 Years

**DOI:** 10.1371/journal.pone.0122216

**Published:** 2015-04-07

**Authors:** Jie Xu, Dasen Li, Lu Xie, Shun Tang, Wei Guo

**Affiliations:** Musculoskeletal Tumour Centre, People’s Hospital, Peking University, Beijing, China; Johns Hopkins University, UNITED STATES

## Abstract

**Background:**

Mesenchymal chondrosarcoma(MCS) is a rare high-grade variant of chondrosarcoma. Consensus has not been reached on its optimal management. Resection with wide margins is usually recommended, but the effect of margins has been demonstrated by little positive evidence. Moreover, the effectiveness of adjuvant chemo- and/or radiotherapy remains controversial.

**Objectives:**

To describe the clinical characteristics and outcomes of MCS of bone and soft tissue, to assess the efficacies of surgery, chemotherapy and radiation, and finally to deliver a more appropriate therapy.

**Materials and Methods:**

We reviewed EMBASE-, MEDLINE-, Cochrane-, Ovid- and PubMed-based to find out all cases of MCS of bone and soft tissue described between April 1994 and April 2014. Description of treatment and regular follow-up was required for each study. Language was restricted to English and Chinese. Issues of age, gender, location, metastasis, and treatment were all evaluated for each case. Kaplan-Meier Method and Cox Proportional Hazard Regression Model were used in the survival analysis.

**Results:**

From the 630 identified publications, 18 meeting the inclusion criteria were selected, involving a total of 107 patients. Based on these data, the 5-, 10-and 20-year overall survival are 55.0%, 43.5% and 15.7% respectively. The 5-, 10-, 20- year event-free survival rates are 45.0%, 27.2% and 8.1%, respectively. Treatment without surgery is associated with poorer overall survival and event-free survival. Negative surgical margins could significantly bring down the local-recurrence rate and are associated with a higher event-free survival rate. Chemotherapy regime based on anthracyclines does not benefit the overall survival. The addition of radiation therapy is not significantly associated with the overall or event-free survival. However, we recommend radiation as the salvage therapy for patients with positive margin so as to achieve better local control.

**Conclusions:**

This review shows that surgery is essential in the management of MCS of bone and soft tissue. Appropriate adjuvant therapy may reduce local recurrence, but cannot benefit the overall survival.

## Introduction

Mesenchymal chondrosarcoma(MCS) is a rare high-grade of variant of chondrosarcoma first described in 1959[1]. It only accounts for 1% to 10% of all chondrosarcomas[2–4]. Histologically, MCS has a typical biphasic pattern consisting of both small cells and islands of atypical cartilage.[4]

MCS differs from typical chondrosarcomas in the following respects. First, MCS has a slight female preponderance and occurs in the nervous system in patients at age 20 to 30 and in soft tissues in patients at age 40 or older, whereas classical chondrosarcomas show a predilection for middle-aged to elderly males.[5] Second, MCS tends to be more aggressive, with 5- and 10-year survival rates of 54.6% and 27.3%, respectively.[6] Although these tumors originate in the bone in most cases, strikingly 22% to 50% of them originate in the soft tissues [7, 8], especially the brain and the meninges [9]. MCS has a high propensity to metastasize to the lungs, lymph nodes, and other bones [6, 8].

For tumors originating from other sites such as the kidney, mandible, orbit, and central nervous system (CNS), the treatment and prognosis are different from tumor originating from bone or soft tissue location. For example, intracranial MCS induces a mortality rate of 54% according to a systematic review involving 60 patients published in 2009[9]. There are less than 500 cases of MCS published, while most of them were reported in case repots or case series. Except one study published in 2014[10], all 3 other series encompassing more than 20 individuals each were published more than 20 years ago [6, 7, 11].

Owing to its rarity, MCS remains poorly understood. Resection with wide margins is usually recommended, but is frequently unfeasible because of anatomic constraints, especially for tumors in the axial regions. MCS is believed to be more sensitive to chemotherapy and radiation compared with other types of chondrosarcomas. The systematic therapy for MCS may follow Ewing sarcoma as recommended in National Comprehensive Cancer Network (NCCN) and European Society for Medical Oncology (ESMO) guidelines. However, most reports on MCS are case reports or retrospective case series. Is MCS really sensitive to adjuvant therapy? We tried but failed to find strong evidence. Therefore, the effectiveness of adjuvant chemo- and/or radiotherapy (ACT/ART) remains controversial [2, 3, 6, 10–13].

The objectives of this systematic review are to describe the prognosis of MCS in bone and soft tissue, to assess the efficacies of surgery, chemotherapy and radiation, and finally to deliver a more appropriate therapy.

## Methods

### Search Strategy

A search was performed in EMBASE, MEDLINE, Ovid, PubMed and Cochrane Library (Cochrane database of systematic reviews, database of abstracts of reviews of effects, and Cochrane central register of controlled trials) to identify studies (including case reports and case series published between 1994 and 2014) evaluating the treatment of MCS in bone and soft tissue. The databases were searched using a combination of the following items: “mesenchymal” and “chondrosarcoma”.

### Eligibility Criteria

We included all cohort studies about MCS, including randomized controlled trials, case series and case reports. Each study had to report original data on age, gender, location, metastasis, treatment with surgery/chemotherapy/radiation, and follow-up period. The inclusion criteria are: (i) case series design with more than one case reported, (ii) follow-up data available for all patients (at least 3 months); (iii) information for the data from each patient to be completely disaggregated. The exclusion criteria are: (i) all MCS originating from sites other than bone and soft tissues were excluded; (ii) patients dead within3 months after diagnosis or without any treatment.

### Study Selection

To identify potentially relevant studies, two authors (XJ and LDS) independently evaluated the titles and then the abstracts on the basis of the eligibility criteria. Full-text articles published in English or Chinese were screened to test the eligibility. The reference lists of these articles were also searched to find out additional articles. Among patients with MCS arising in bones or soft tissues, only those with adequate histologic proof of the disease and satisfactory data concerning treatment and follow-up were included in this analysis. Disagreement was solved through discussion.

### Data Extraction

One author extracted the data using a standardized form, while the second author checked the extracted data. The following data were collected: demographic and clinical characteristics, location, therapy, outcomes after consecutive therapy, and the duration of follow-up. If more than one published report of the same group of patients, the articles were analyzed to verify whether or not they reported different outcomes. If they presented the same outcomes we extracted the data from the most recent or most complete article. When important data were missing in some studies, the first author (XJ) tried to contact the authors to obtain the additional information. If it was impossible to obtain from the authors, these data were considered missing.

### Assessment of quality

The methodological quality of these studies was assessed by two authors (XJ and LDS) independently, using Grading Quality of Evidence and Strength of Recommendations (GRADE) published in 2004[14]Disagreement was solved through discussion.

### Survival analysis

From these selected studies, only patients with tumors in bone and soft tissue were included in the following analysis. Statistics were calculated on SPSS 17.0 (Chicago, Ill). The survival analysis was conducted using the Kaplan-Meier method and Cox proportional hazards regression. Overall survival (OS), event-free survival (EFS) and recurrence-free survival (RFS) rates were calculated using the Kaplan-Meier estimator. OS was estimated during the time from diagnosis to death, or from therapy, to last follow-up. EFS was estimated during the time from diagnosis to first recurrence or progression (any evidence of growth of a tumor that was not in clinical CR) or last follow-up. If no event occurred, the survival data were censored at last follow-up. [12]Clinicopathological variables described above in data extraction were analyzed with the Cox regression model.

## Results

### Study selection

**Fig 1:**
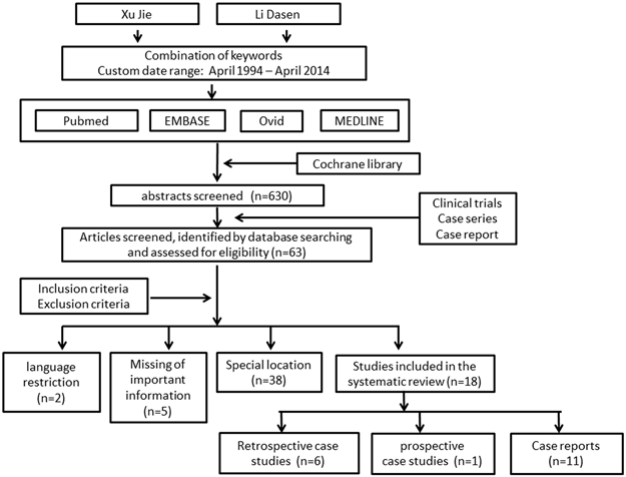
Flow chart shows the process of article selection for this study.

With these key words, the literature search yielded 630 potentially eligible studies. After initial screening, 63 studies (including case reports, retrospective and prospective studies) were manually sorted to extract all descriptions of patients. Among these articles, two were written in other language; important information was unavailable in 5 articles; special locations were found such as CNS in other 38 articles.(Table 1). Finally, 18 articles (Table 2) involving 107 patients with MCS in bone and soft tissue were included in the systematic review (Fig 1), including 11 isolated case reports[15–25] (1/12 involving two cases[26]), 6 retrospective case studies involving 88 patients [2–4, 10, 13, 27] and 1 prospective study involving 6 patients[12]. The description of all articles is displayed in Table 2. Among the 18 papers, 16 were published in English (n = 104), and two were in both English and Chinese (n = 3). Owing to the different biological behaviors and treatments, tumors at special locations were excluded, including skull, CNS, kidney, infant, eye, orbit, lung, spleen, breast, heart, thyroid, sinonasal tract, jaws, gastrointestinal tract, mediastinum, uterus and vulva.

**Table 1:** Studies excluded from this review.

No	Authors	Source	Reason
1.1	Solov'ev IuN	Vopr Onkol. 1983;29(9):21–6[37]	Article in Russian
1.2	Heyer CM, Roggenland D	Pneumologie. 2007 Feb;61(2):94–8.[38]	Article in German
2.1	Italiano A	Ann Oncol. 2013 Nov;24(11):2916–22. [39]	Lack of important clinical information
2.2	Jambhekar NA	Indian J Pathol Microbiol. 2004 Oct;47(4):491–3.[40]	Didn’t have enough information for the data from each patient to be completely disaggregated
2.3	Jain M, Puri V, Madan NK.	Indian J Med Sci. 2011 Dec;65(12):552–6[41]	follow-up data unavailable
2.4	Ramraje SN, Kulkarni SS.	Australas Med J. 2011;4(8):448–50.[42]	follow-up data unavailable
2.5	Johnson DB, Breidahl W	Skeletal Radiol. 1997 Aug;26(8):501–4.[43]	follow-up data unavailable
3.1	Nguyen DV, Muda AS	Malays J Med Sci. 2013 May;20(3):71–7.[44]	Tumor in skull
3.2	Hu HJ, Liao MY, Xu LY.	Oncol Lett. 2014 Jun;7(6):1970–1974. [45]	Tumor in vena cava
3.3	Jeh EA1, Lee YJ, Kim HY	Obstet Gynecol Sci. 2013 Sep;56(5):345–8.[46]	Tumor in vulva
3.4	Khouja N1, Ben Amor S	Surg Neurol. 1999 Jul;52(1):50–3.[47]	Tumor of the orbit
3.5	Ram H1, Mohammad S	J Maxillofac Oral Surg. 2011 Dec;10(4):340–3. [48]	Tumor of mandible
3.6	Glien A1, Moser O, Göke F	HNO. 2012 Dec;60(12):1086–90. [49]	Tumor of the lateral skull base
3.7	Hanakita S1, Kawai K	Neurol Med Chir (Tokyo). 2012;52(10):747–50.[50]	Tumor of the orbit
3.8	Xu H, Shao M, Sun H, Li S.	Diagn Pathol. 2012 Sep 21;7:125. [51]	Tumor in kidney
3.9	Herrera A, Ortega C	Case Rep Med. 2012;2012:292147. [52]	Tumor of the orbit
3.10	Kan Z	Br J Neurosurg. 2012 Dec;26(6):912–4. [53]	Intracranial tumor
3.11	Cheim AP Jr	J Oral Sci. 2011 Jun;53(2):245–7.[54]	Tumor in the mandible
3.12	Rossetto A	Tumori. 2011 Jul-Aug;97(4):e10-5. [55]	Tumor of the spleen
3.13	Sardi I, Massimino M	Pediatr Blood Cancer. 2011 Apr;56(4):685–6. [56]	Intracranial tumors
3.14	Liu M1, Qin W, Yin Z	Clin Imaging. 2010 Sep-Oct;34(5):379–81.[57]	Tumor of the orbit with intracranial extension
3.15	Razak AR	Eur J Cancer Care (Engl). 2010 Jul;19(4):551–3. [58]	Mesenchymal chondrosarcoma of the orbit
3.16	Misra V1, Singh PA	Acta Cytol. 2008 May-Jun;52(3):366–8.[59]	Tumor of meninges
3.17	Kaur A	Orbit. 2008;27(1):63–7. [60]	Mesenchymal chondrosarcoma of the orbit
3.18	Belhachmi A	J Neuroradiol. 2008 Jul;35(3):189–91. [61]	Spinal intradural mesenchymal chondrosarcoma
3.19	Odashiro AN	Int Ophthalmol. 2009 Jun;29(3):173–7. [62]	Primary orbital mesenchymal chondrosarcoma
3.20	Hsing CT	Cancer Res Treat. 2007 Sep;39(3):131–3. [63]	tumor of the heart
3.21	Pellitteri PK	Oral Oncol. 2007 Nov;43(10):970–5. [64]	Mesenchymal chondrosarcoma of the head and neck
3.22	Bencheikh R	Rev Stomatol Chir Maxillofac. 2007 Apr;108(2):156–8. [65]	Tumor of the mandible. Article in French
3.23	Angotti-Neto H	Ophthal Plast Reconstr Surg. 2006 Sep-Oct;22(5):378–82.[66]	Tumor of the orbit.
3.24	Kaneko T	Int J Urol. 2006 Mar;13(3):285–6.[67]	Tumor of the kidney
3.25	Chen SH	Acta Paediatr Taiwan. 2005 Sep-Oct;46(5):308–10.[68]	Intraspinal tumor
3.26	Hamada H	Otolaryngol Head Neck Surg. 2005 Oct;133(4):639.[69]	Tumor of of the nasopharynx
3.27	Yassa M	J Neurooncol. 2005 Sep;74(3):329–31.[70]	Intra-parenchymal tumor
3.28	Salvati M	J Exp Clin Cancer Res. 2005 Jun;24(2):317–24.[71]	Tumor of Central nervous system
3.29	Abbas M	APMIS. 2004 Jun;112(6):384–9.[72]	Tumor of the thyroid
3.30	White DW	Clin Imaging. 2003 May-Jun;27(3):187–90.[73]	Tumor of the retroperitoneum
3.31	Knott PD	Laryngoscope. 2003 May;113(5):783–90.[74]	Tumor of the sinonasal tract
3.32	Inenaga C	Acta Neurochir (Wien). 2003 Jul;145(7):593–7[75]	Tumor of the sellar region
3.33	Huang KF	Chang Gung Med J. 2003 May;26(5):370–6.[76]	Primary intraspinal tumor
3.34	Huang HY	Ann Thorac Surg. 2002 Jun;73(6):1960–2.[77]	Primary mesenchymal chondrosarcoma of the lung
3.35	Kashyap S	Orbit. 2001 Mar;20(1):63–67.[78]	Tumor of the orbit
3.36	Gomez-Brouchet A	J Urol. 2001 Dec;166(6):2305.[79]	Tumor of the kidney
3.37	Nesi G	Ital Heart J. 2000 Jun;1(6):435–7.[80]	Tumor involving the heart
3.38	Bingaman KD	Neurosurgery. 2000 Jan;46(1):207–11; [81]	Intracranial tumor

**Table 2:** General characteristics of the included studies.

No	Authors	Year	Source	Language	Study design	GARDE	N total	N	Impact factor
1	Tsukamoto et al.[15]	2014	Case Rep Oncol Med	English	case report	low	1	1	2.147
2	Kawaguchi, S., et al.[10]	2014	Clin Orthop Relat Res	English	retrospective case series	low	43	30	2.787
3	Seo, C.Y[27]	2012	Korean J Pathol	English	retrospective case series	low	2	2	0.174
4	Shakked, R.J., et al.[2]	2012	Arch Pathol Lab Med	English	retrospective case series	low	20	10	2.781
5	Kupeli, S., et al[16]	2010	Pediatr Hematol Oncol	English	case report	low	1	1	0.895
6	Zibis, A.H.[17]	2010	Clin Orthop Relat Res	English	case report	low	1	1	2.787
7	Dantonello, T.M.,et al[12]	2008	Cancer	English	prospective case report	high	15	6	5.201
8	Cesari, M., et al[3]	2007	Tumori	English	retrospective case series	low	26	26	0.922
9	D'Andrea, G., et al.[18]	2008	Neurosurg Rev	English	case report	low	1	1	1.972
10	Zhao, F., et al.[19]	2007	Foot Ankle Int	English	case report	low	1	1	1.474
11	Cai, L., Z.F[26]	2006	Beijing Da Xue Xue Bao	Chinese	case report	low	2	2	0.760
12	Anderson, J.T.[20]	2007	J Hand Surg Am	English	case report	low	1	1	1.572
13	Matsuda, Y., et al.[21]	2006	Spine	English	case report	low	1	1	2.159
14	Zhang, J.Y., et al.[22]	2006	Zhonghua Bing Li Xue Za Zhi	Chinese	case report	low	1	1	0.612
15	Amukotuwa, S.A.et al[23]	2006	Skeletal Radiol	English	case report	low	1	1	1.741
16	Hashimoto, N., et al[13]	2005	Skeletal Radiol	English	retrospective case series	low	10	10	1.741
17	Nussbeck, W., et al.[4]	2004	Pathology	English	retrospective case series	low	10	9	2.657
18	Aoki, T., et al.[24]	1996	Surg Today	English	case report	low	1	1	0.963

N total: number of total patients in the study

N: number of patients located in bone and soft tissue

### Studies and patients Characteristics

Totally 107 patients with MCS in bone and soft tissue were finally included in this review. Their demographic and clinical characteristics are described in Table 3. There are 56% males and 44% females, with more than three quarters under 40 years old. The ratio of locations between bone and soft tissue is 3:2, which is the same to extremities and trunk. Primary tumor sites were thigh (n = 32), calf (n = 14), upper arm (n = 14), forearm (n = 3), spine or paraspine (n = 13), pelvis (n = 17), rib or chest wall (n = 13), and toes (n = 1). The treatments, including surgery (n = 99; 92.7%), chemotherapy (64; 63.4%), and radiation (28; 27.7%). About 43(39.8%) patients were treated with both surgery and chemotherapy, 7(6.5%) patients with both surgery and radiation, and 16(14.8%) patients with all three therapies.

**Table 3:** Demographic and clinical characteristics (Total patients = 107).

	N	%
Sex		
male	47	56
female	37	44
Age(yr)		
0–10	2	2
10–20	18	22
20–30	30	36
30–40	14	17
40–50	10	12
>50	9	11
**Location**		
*Category 1*		
trunk	43	41
Extremities	64	59
*Category 2*		
bone	63	60
Soft tissue	42	40
Metastasis		
Local	73	75
metastasis	24	25
treatment		
S	36	33
S+C	43	40
S+R	7	6
S+C+R	16	15
C	1	1
C+R	4	4
R	1	1

S, surgery; C, chemotherapy; R, radiation

### Statistical analysis

The minimum follow-up period is 4 (mean 48, range 4–288) months, and the median EFS is 57 months. The 5-, 10-, 15,-and 20-year EFS rates are 45.0%, 27.2%, 16.2% and 8.1%, respectively. The 5-, 10-, 15-, and 20 year OS rates are 55.0%, 43.5%, 35.4%and 15.7%, respectively.

**Fig 2:**
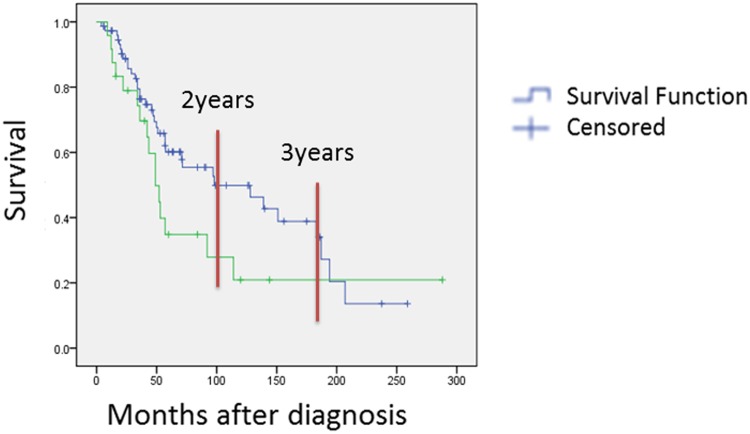
Comparison of overall survival rate in local disease and metastatic disease.

Unlike other types of sarcomas such as osteosarcoma and Ewing sarcoma, patients with MCS are not significantly different in OS whether they suffer from local disease or metastatic disease upon diagnosis (*p* = 0.095)(Fig 2). It seems that in the first 2 years, the OS rate is higher in local disease than in metastatic patients. However, when patients successfully survived more than 3 years after diagnosis, the OS rates of the two groups converged. This result may be expressed by its high metastatic rate. Subgroup analysis was performed involving 76 patients with localized disease. Among these patients, the 5 and 10-year EFS rates are 49.1% and 31.2%, respectively (median EFS period 57.5 months), and the 5 and 10-year OS rates are 54.8% and 38.3%, respectively (median OS period 80.0 months).

**Fig 3:**
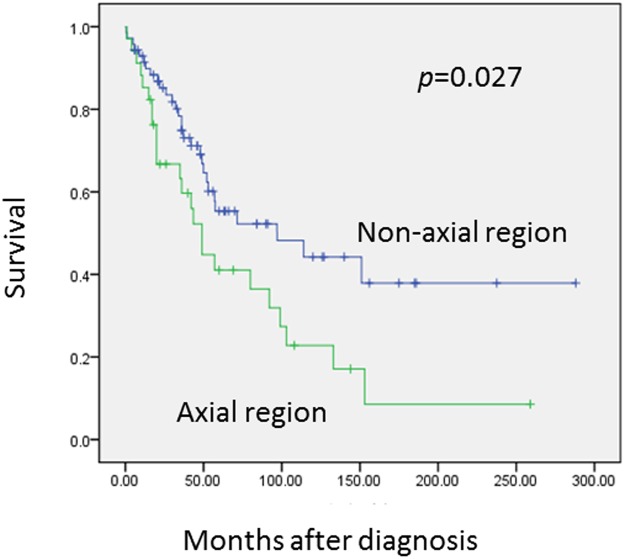
Compared with non-axial region, tumor in axial region has a much lower overall survival rate (*p* = 0.027).

**Fig 4:**
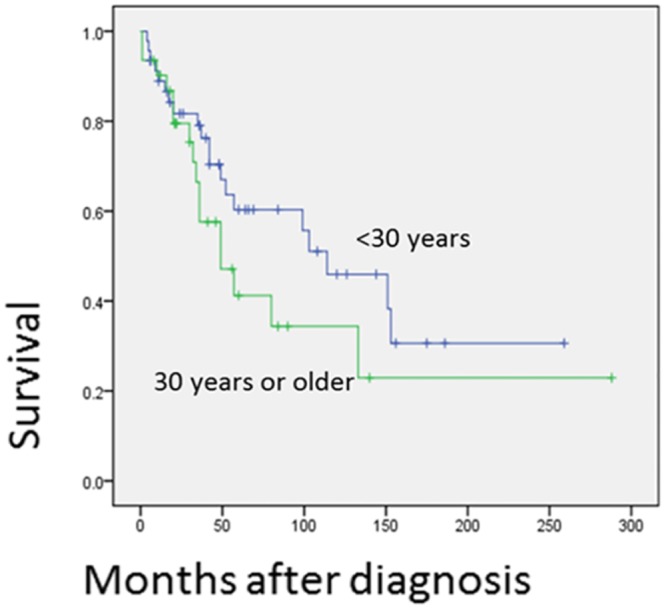
Prognostic significance of age of the patients is shown. It is comparison of patients elder than 30 and those younger than 30.

**Fig 5:**
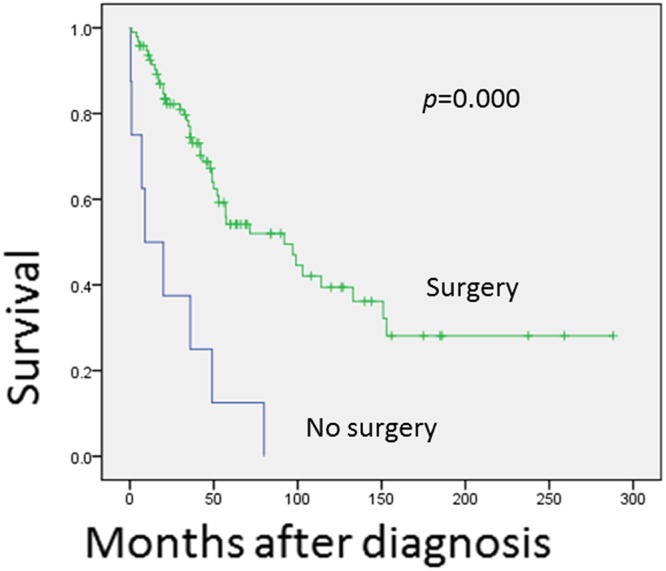
This figure shows the comparison of overall survival for patients treated with surgery and those without. A statistically significant difference was noted between the groups by the COX regression test (p = 0.000).

**Fig 6:**
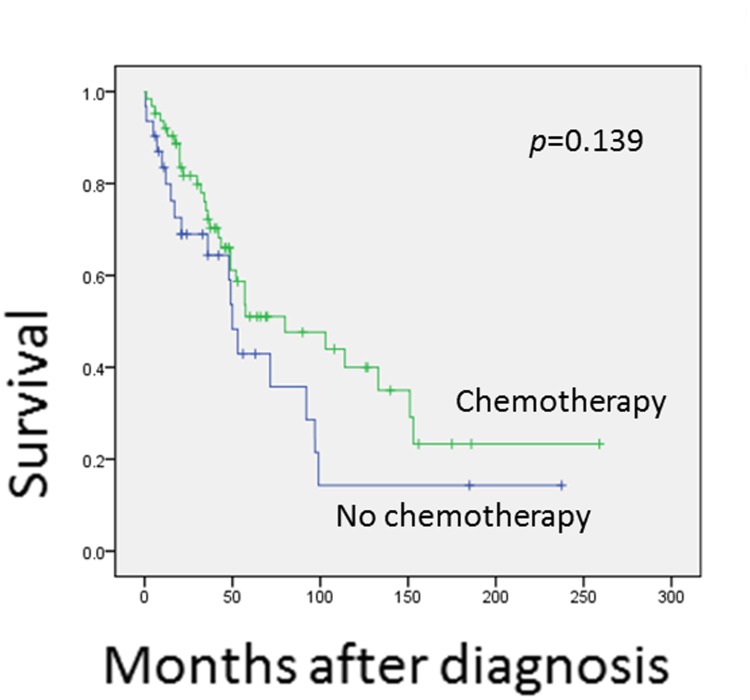
This figure shows the comparison of overall survival for patients treated with chemotherapy and those without. There was a trend for patients to get a better overall survival with chemotherapy, however with no statistically significany.

Univariant analysis shows that tumors in the axial region are associated with a worse prognosis (*p* = 0.027)(Fig 3). The 5 and 10-year OS rates for the axial region are 41.0% and 22.8%, respectively, but are 55.3% and 44.2%, respectively, for non-axial regions. This difference may be explained by the difficulty in reaching wide surgical margins in the axial region. Patients at age below 30 years tend to have a decreased OS rates (Fig 4), but not significantly (*p* = 0.173). Treatment with surgery is significantly associated with improved OS (*p* = 0.000)(Fig 5) and EFS (*p* = 0.000). In contrast, the treatment of chemotherapy would benefit EFS (*p* = 0.046) not OS (*p* = 0.139)(Fig 6). None of gender, origin and site of the tumor (trunk vs. extremities; bone vs. soft tissue), or radiation is significantly associated with OS or EFS. Patients treated with radiation tend to show a lower recurrence rate, but not significantly (*p* = 0.199).

**Fig 7:**
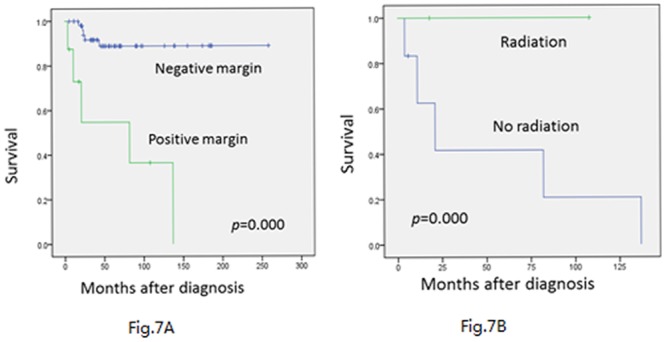
Local-recurrence-free survival of these 82 patients was estimated using the Kaplan-Meier plots. (A) A statistically significant difference was noted between the positive margins and negative margins by COX regression (*p* = 0.000). (B) For positive margins, postoperative radiation therapy could remarkably bring down local recurrence rate(*p* = 0.013).

Surgical margins are important in the management. Only 82 of the 107 patients were able to provide information of surgical margins. Moreover, 67 patients received wide or radical resection and got a negative margin, while 15 patients underwent intralesional surgery and got a positive margin. Negative margins are significantly associated with improved local-RFS (*p* = 0.000)(Fig 7A) and EFS(*p* = 0.050). For 15 patients with positive margins, 1 of them was lost to follow-up. For the 14 patients, 5 of them received postoperative radiation as the salvage therapy, and only 2/5 of them experienced local recurrence. For the other 9 patients without receiving radiation therapy, the local recurrence rate is up to 90% (8/9). The Cox regression demonstrates that postoperative radiation can significantly bring down the local recurrence rate for patients with positive margins (*p* = 0.013)(Fig 7B). However, postoperative radiation doesn’t benefit the OS and EFS rates in this group. About 10/14 patients received chemotherapy after surgery, but it did not benefit the survival (*p* = 0.436).

## Discussion

MCS is a rare high-grade variant of chondrosarcoma. Because of its rarity, most studies about MCS only involve a small sample size, and there are few clinical trials involving more than 10 patients in the past 20 years [10, 12]. Tumors originating from special sites, such as CNS, are usually associated with special prognosis and treatment. This systematic review summarizes the available evidences on MCS in bone and soft tissue in the last 2 decades, and represents the survival condition and assesses the efficacies of surgery, chemotherapy and radiation.

Of the 630 potentially eligible articles, only 19 studies, with low to high quality meet the inclusion criteria. The pooled results (from 19 studies) show that the 5 and 10-year OS rates were 55.0% and 43.5%, respectively. The OS rates has slightly improved compared with studies of MCS (N>10) published 20 years ago [6, 7, 11, 28, 29] (Table 4).

**Table 4:** Studies (N>10) of MCS published 20 years ago.

author	Year	source	N total	N	Averageage	5yOS%	10yOS%	5yEFS %	10yEFS%
Salvador et al.[11]	1971	cancer	30	19	28.4	ND	ND	ND	ND
Harwood et al.[29]	1980	CORR	17	12	29.4	ND	ND	ND	ND
Dabska and Huvos[28]	1983	Virchows Arch	19	ND	<21	35	20	ND	ND
Huvos et al.[7]	1983	cancer	35	30	24.7	42	28	ND	ND
Nakashima et al.[6]	1986	cancer	23	ND	ND	55	27	ND	ND
Our study	-	-	110	110	29.5	50	35	45	27

ND = not described

N = number of patients with MCS in bone and soft tissue

Recently, studies in MCS focus on specific sites such as jaws [30], CNS [8], skull and sinonasal tract [31], which were reported to have different outcomes. It is still open to discussion whether the specific anatomic sites result in milder histological behavior, smaller size at diagnosis, different therapy and finally a better outcome [8, 9, 30, 31]. Our systematic review focuses on the classical sites of bone and soft tissue, and excludes those arising from special anatomic locations, thus guaranteeing the heterogeneity of this study and the following survival analysis.

on the result from long-term follow-up suggests that the careful monitoring after diagnosis should last more than 10 years, because MCS has a strong tendency toward late local and metastatic recurrence. Estimation of conditional survival (CS) for cancer patients diagnosed at different ages and disease stages provides patients and clinicians with important information in planning of follow-up, surveillance and management. For most of cancers, patients usually have a much higher chance to survive another 5 years once they have successfully survived 5-years or more on their cancer journey [32]. The 5-year CS can be estimated using Paul Dickman’s method [33] for period analysis. The 5-year CS rate for osteosarcoma and Ewing sarcoma [34] can reach up to 91.4% if the patients have already survived 10 years. However, our analysis shows that MCS patients still suffer from a poor 5-year survival rate of less than 50% even after a survival of 10 or even 15 years. It reminds us that continuous monitoring is needed even 15 years after diagnosis.

Wide local excision to achieve a wide surgical margin is commonly regarded as the “gold standard” in treatment of MCS. But little positive evidence has been obtained to demonstrate the effect of margin perhaps owing to the rarity of MCS [10, 12]. The importance of adequate surgery has been confirmed in a study involving 26 patients in a single institute in Italy, since all the 5 patients with positive margins died in the follow up, which suggests that margins should be the first goal [3]. To our best knowledge, no previous study shows a significantly association between surgical margins and the final survival rate, including RFS and EFS. Our finding demonstrates the importance of clear margin in treatment of MCS for the first time.

In the terms of radiation, it seems to only benefit patients with positive margins. A retrospectively review involving the cases of MSC diagnosed between 1979 and 2010 at MD Anderson Cancer shows that the addition of ART for MCS would benefit local control of MCS (no radiated patients experienced local recurrence during the follow-up period, while nearly 70% of the in-radiated patients suffered from [10]. However, this excellent local control failed to show the advantage in EFS or OS, which meant that patients escaping from local recurrence had the same risk of metastasis or death because of this malignancy. This result coincided with our study. Although patients with positive margins may escape from local recurrence within half a year without postoperative radiation [19], we still recommend that for patients who are unable to get radical resection, irradiation is an effective remedial measure to reduce the recurrence rate.

Highly variable regimes are used as ACT of MCS, including dactinomycin (AMD), carboplatin (CAR), cisplatin (DDP), cyclophosphamide (CYC), doxorubicin (DOX), etoposide (ETO), ifosfamide (IFO), high-dose methotrexate (MTX), and vincristine (VCR). In most cases, they are used in different combinations or following Ewing’s sarcoma regimes, and DOX seems to be essential in all types of regimes [2, 3, 6, 7, 11–13, 21, 28, 29]. Patients with MCS can be divided by the histologic categories into two groups to select a treatment protocol [7]. Those exhibiting a small cell pattern received an ACT regime consulting Ewing family of tumors (focusing on DOX and CTX), while the hemangiopericytomatoid pattern received a reported regime in treatment of osteosarcoma. Using this histological specific regime (also the unique one), the OS rate was 42% at 5 years and 28% at 10 years. However, some doctors are skeptic to this histologic specific regime, since the overlap of histologic patterns commonly appears in some specimens [6]. High p-SMAD2 and PAI-1 expressions have been found in MCS specimens, which points to the importance of TGFb signaling as a promising therapeutic option. Targets on the Bcl-2 family members may also be effective [35]. In summary, there is no general agreement on the regime of chemotherapy as the adjuvant therapy of MCS, except for DOX as a cornerstone. However, it must be stressed that the majority of patients relapse with distant metastases, which indicates the importance of using an effective systemic control group.

The ambiguous response to chemotherapy or radiation may relate to the specific histology of MCS. MCS contains a typical biphasic pattern of undifferentiated small round cells, which resemble the Ewing family of tumors and are blamed to be the highly malignant part, blended with islands of well-differentiated hyaline cartilage [36]. For an individual patient with MCS, the crucial factors probably affecting his/her outcome include the proportion of spindle-cell or round-cell elements and cartilage, the pattern of distribution, and the transition between these two parts. On the other hand, MCS can be separated by histologic examination into two subgroups: those with a predominant hemangiopericytomatoid growth component, and those with a small cell undifferentiated cellular pattern [7]. The latter is usually more sensitive to drugs and radioactive rays, but presents a higher malignant behavior and a worse outcome. From this perspective, MCS of this pattern may benefit more from chemotherapy.

Because of the rarity of MCS, most of the studies in our review are case reports or small-size case series. It is difficult to get uniform criteria in the surgical margin, the regime in chemotherapy and radiation among different studies. It remains to be determined whether lack of a significant impact of chemotherapy over the survival rates is the result of limited efficacy of the chemotherapy or the selection bias of the patients.

### Implications for Clinical Practice and Research

Our study demonstrates that MCS has a strong trend toward late local and metastatic recurrence. A long-term surveillance of more than 20 years is necessary in the management. Surgery is significantly associated with improved OS and EFS rates. Radical surgery with negative surgical margin, if possible, seems to be the mainstay therapy for MCS. In contrast, the treatment with chemotherapy does not benefit OS. Radiation itself fails to show significant association with either OS or EFS. This result reminds us that not every MCS patient will benefit from aggressive therapy. We recommend that for lesions not amenable to ablative surgical treatment, irradiation is an effective remedial measure to bring down the recurrence rate. For patient with more malignant MCS in pathological behavior, the addition of adjuvant chemotherapy may play a more important role. More effective regimes and new targeted therapy are needed in the future. The differentiation of the Ewing-like pattern from the hemangiopericytoma-like pattern in the spectrum of MCS may attract more interest from a therapeutic standpoint.
